# Visual impairment and associated factors among pregnant women attending antenatal care units at health institutions in Gondar City Administration, Northwest Ethiopia

**DOI:** 10.1186/s12884-021-04302-6

**Published:** 2021-12-13

**Authors:** Mengistie Diress, Yitayeh Belsti, Mihret Getnet, Sofonias Addis Fekadu, Baye Dagnew, Yonas Akalu, Mohammed Abdu Seid, Yibeltal Yismaw Gela

**Affiliations:** 1 Department of Human Physiology, School of Medicine, University of Gondar, P. O. Box 196, Gondar, Ethiopia; 2 Department of Optometry, School of Medicine, University of Gondar, Gondar, Ethiopia; 3 Unit of Human Physiology, Department of Biomedical Sciences, School of Health Science, Debre Tabor University, Debre Tabor, Ethiopia

**Keywords:** Visual impairment, Presenting visual acuity, Pregnancy, Ethiopia

## Abstract

**Background:**

Visual impairment is a major public health concern among women of reproductive age groups in Ethiopia, which is getting worse during pregnancy. Though visual impairment has lots of serious consequences across the life course of pregnant women, there is no previous study on this topic in Ethiopia. Thus, this study determined the prevalence of visual impairment and identified associated factors among pregnant women attending antenatal care units at the governmental health institutions in Gondar City Administration, Northwest Ethiopia**.**

**Methods:**

An institution-based cross-sectional study was conducted. A systematic random sampling technique was used to recruit the study participants. We used an interviewer-administered questionnaire comprising of socio-demographic, clinical and pregnancy-related variables to collect the required data. Snellen’s illiterate “E” chart was used to determine visual impairment. EpiData 3 and Stata 14 were used for data entry and statistical analysis, respectively. Both bivariable and multivariable binary logistic regression analyses were executed to identify associated factors of visual impairment. Variables with a *p*-value ≤0.05 in the multivariable logistic regression analysis were declared as statistically significant with visual impairment.

**Results:**

A total of 417 (response rate = 98.6%) participants were involved in this study, with a median age of 27 years. The overall prevalence of visual impairment was 22.5% (95% CI: 18.5–26.6). Thirty (7.2%) and thirty-two (7.7%) of the study participants had moderate to severe visual impairments in their right and left eyes, respectively. Participants aged from 31 to 49 years (AOR = 2.1; 95% CI: 1.1–4.0), being 3rd trimester (AOR = 2.4; 95% CI: 1.3–4.5), multi & grand multipara (AOR = 2.3; 95% CI: 1.2–4.6), and history of contraceptive use (AOR = 2.7; 95% CI: 1.2–6.3) had higher chance of **v**isual impairment.

**Conclusion:**

The magnitude of visual impairment among pregnant women was high in the study area. Therefore, routine screening and evaluation of pregnant women for visual condition during antenatal care visits is recommended. Further investigations of visual changes, particularly as a result of pregnancy, are warranted.

## Introduction

Visual impairment (VI) is a state in which one or more functions of the visual system are troubled due to physiological or pathological disorders aroused from either or both eyes [1]. According to the revised definition by the World Health Organization (WHO), VI is defined as the presenting distance visual acuity (PVA) worse than 6/12 in the better eye. It is classified as mild (PVA < 6/12 to ≥6/18), moderate (PVA < 6/18 to ≥6/60), severe (PVA < 6/60 to ≥3/60), and blind (PVA < 3/60) in the better eye [1, 2].

Globally, about 2.2 billion people have been suffered by vision impairment, which is more worryingly in developing countries, among elderly people, females, and rural communities [1, 3]. Based on a systematic review and meta-analysis in 2017, females are highly affected by VI [3, 4]. Another study reported that the incidence of VI among women is 55% (139 million) out of 253 million people in the world who are visually impaired [3]. The prevalence of vision loss among women in sub-Saharan Africa is 4.17% [5]. Based on the national survey in Ethiopia, mild and moderate to severe visual impairment (MSVI) are more prevalent among females which accounts 4.1% [6].

During pregnancy, metabolic and hormonal changes can upset the normal visual functions of the women’s eyes. Vision impairment is a chief complaint of most women during pregnancy. This problem is due to either physiological changes or exacerbations of pre-existing medical conditions [7–9]. Most ocular changes occurred in pregnancy are temporary but occasionally lead to permanent complications that may affect the health of the women [7, 9]. In Iran, the visual problem was observed in 89.2% of pregnant women which is worse in the third trimester of gestation [8]. Another study in Nigeria showed that VI due to refractive error is the common problem during pregnancy though most of them are transient [7]. According to a recent study in Ethiopia, 35.66% of pregnant women had a refractive error, which can be one cause of VI [10]. Retinal change, which could result in VI in pregnant women with pregnancy-induced hypertension is about 12% [11]. The prevalence of VI among pregnant women with pre-existing medical conditions is 25 to 50% [12].

Visual impairment can lead to several potential consequences throughout the life course of pregnant women. If not treated early, it will increase the risk of blindness, decrease the general well-being of pregnant women and even lead to death [13]. It can also reduce productivity, increase the risk of depression and social loneliness, lead to an inability to perform tasks alone, increase the risk of fall-associated injuries, and sexual violence and abuse [1].

According to the previous studies in the globe, VI has a cross relation with the age of pregnant women [1, 14, 15], residence [1, 16], educational status [17–19], occupation [20, 21], gestational age (GA) [7, 15, 22], maternal parity [15, 20, 23], diabetes mellitus [24, 25], gestational diabetes mellitus (GDM) [14, 20], pre-existing hypertension [12, 20], pregnancy-induced hypertension (PIH) [14, 20], family history of vision problem [26], prolonged use of smart phones and computers [27–29], medication history [14, 30, 31], and history of contraceptive use [14, 32].

Even though VI has lots of serious consequences across the life course of pregnant women, there is no previous study in Ethiopia on this topic. Thus, this study aimed to determine the prevalence of VI and associated factors among pregnant women attending antenatal care units at selected governmental health institutions in Gondar City Administration. Knowledge on the prevalence of ocular problems among pregnant women can help clinicians and policymakers to design appropriate prevention strategies.

## Methods and materials

### Study design, setting, and population

An institution-based cross-sectional study design was employed from 15 October to 15 December 2020. The study was conducted at selected governmental health institutions in Gondar city administration. Gondar is a historical city in Ethiopia located 727 km far from the capital city, Addis Ababa in the Northwest direction. It has 12 sub-cities with 12 urban and 10 rural kebeles. In the city administration, there are eight health centres (HC) and one teaching referral hospital providing antennal care (ANC) services for about 37,000 pregnant women annually. The study was conducted among pregnant women of 15–49 age groups. All pregnant women who visited ANC services of the selected health institutions were included in the study whereas, those with congenital eye problems and eye trauma during the study period were excluded.

### Sample size determination and sampling procedure

**Fig. 1:**
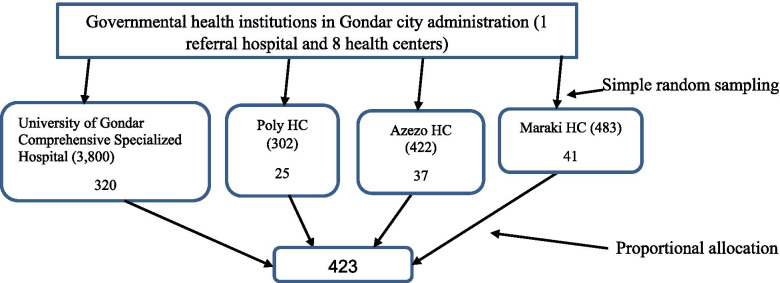
Selection of pregnant women (sample size) visiting ANC services at governmental health institutions in Gondar city administration, Gondar, Ethiopia, 2020

Sample size was calculated using single population proportion formula. There was no evidence within the same study area to estimate the minimum sample size. Therefore, 0.5 proportion of the population with VI, 5% margin of error, 95% confidence interval, and 10% non-response rates were considered to calculate the sample size. Hence, the total sample size became 423. A simple random sampling method was used to select health institutions for this study. Four governmental health institutions (three health centres and one referral hospital) were randomly selected by lottery methods. Study participants were recruited from selected health institutions by a systematic random sampling technique. For the better representativeness of sample size to the source population, the proportional allocation was performed for each institution (Fig. 1).

### Study variables

The dependent variable was visual impairment. The independent variables were age, residence, occupation, educational level**,** parity, gestational age, history of DM, GDM, history of HTN, PIH (preeclampsia and eclampsia)**,** history of khat chewing and cigarette smoking, coffee drink, alcohol intake, medication history, and regular use of smartphones and computers or watching TV, history of contraceptive use, stress**,** sleep disturbance, and family history of vision problem.

### Operational definitions

Visual impairment: the study participants were classified as visually impaired if the presenting VA in the better eye was worse than 6/12 and unless otherwise, normal, if the presenting VA in the better eye was ≥6/12 [1, 2].

Regular use of computers or television: Reading or watching computers or television at least once a day for not less than 2 h.

Regular use of smartphones: Using smartphones at least once a day for more than 2 h.

Sleep disturbance: Sleeping time of ≤5 h/day or sleeping time of ≥9 h/day.

Medication History: Taking anti-rheumatic, anti-psychiatric & anti-thrombotic drugs in the last 30 days.

### Data collection tools, procedure, and quality management

A structured-interviewer-administered questionnaire consisting of socio-demographic, obstetric, and other clinically related variables was used to collect the required data. A pre-test study was conducted among 22 pregnant women outside of the study area prior to the actual data collection. The tool was modified based on the findings from the pre-test results. Some ambiguous questions and words were rewritten for a better understanding of study participants. Reliability was also checked by scale reliability coefficient and the overall Cronbach’s alpha result was 0.73, which is acceptable. Presenting visual acuity test was done in each eye separately using Snellen’s illiterate “E” chart in a well-illuminated room, hanging on a wall at a distance of 6-m. All through the test, pregnant women were sitting or standing 6 m away from the chart and ordered to cover one eye and read out loud the letters they saw with their uncovered eye. The examiner had asked them to read smaller and smaller letters until they can no longer accurately distinguish letters. This procedure was repeated for the other eye and measurements were recorded. Eye examination was performed at a private room in each health institution during the data collection period. Data were collected by three BSc Midwives recruited from three health centers and two Optometrists from the department of clinical optometry at the University of Gondar. The study participants had gotten counseling and a referral system depending on the ocular findings. The training was given to the data collectors and the supervisor about the objectives of the study, data collection techniques and ethical issues. Strict supervision was undertaken during the process of data collection.

### Data processing and analysis procedure

The collected data were entered into EpiData 3.1 and exported into STATA 14 for statistical analysis. Descriptive measures like median, frequency and interquartile range (IQR) were calculated. Bi-variable binary logistic regression analysis was used to select the candidate variables for the final model. Those variables with a *p*-value of < 0.25 in the bivariable binary logistic regression analysis were selected for multivariable binary logistic regression. Multivariable binary logistic regression analysis was executed to identify factors associated with VI. Measure of association was defined by the adjusted odds ratio (AOR) with its 95% confidence interval. In the final model, variables with a p-value ≤0.05 were declared as statistically associated with VI. Model fitness was checked by the Hosmer and Lemeshow goodness of test (at *p* > 0.05) and multi-collinearity was tested by a variance inflation factor (VIF).

## Results

### Socio-demographic characteristics of pregnant women

In this study, four-hundred and seventeen pregnant women participated with a response rate of 98.6%. Three-hundred and ten (74.3%) of the study participants were aged 15–35 years, and the majority of them (82.7%) were from urban residence. The majority of our study participants were housewives by occupation (33.3%) and 37.2% of them had college or university level of education (Table 1).

**Table 1:** Socio-demographic characteristics of pregnant women attending ANC units at governmental health institutions in Gondar city administration, Northwest Ethiopia, 2020 (n = 417)

Variables	Frequency	Percentage (%)
**Age in years**		
15–30	310	74.3
31–49	107	25. 7
**Religion**		
Christian	330	79.1
Muslim	87	20.9
**Residence**		
Urban	345	82.7
Rural	72	17.3
**Educational status**		
Cannot read & write	54	12.9
Primary	95	22.8
Secondary	113	27.1
College/University	155	37.2
**Occupation**		
Government employee	107	25.7
Private employee	84	20.1
Merchant	42	10.1
Housewife	139	33.3
Others*	45	10.8

*Others = farmers, daily workers and unemployed

### Lifestyle, clinical, and obstetric-related characteristics

The majority of the study participants were nulli and primiparous (61.6%) and 64.7% of them had a gestational age of 27–42 weeks. Two-hundred and forty-five (58.7%) pregnant women had a history of regular use of smartphones for more than 2 h per day. Two-hundred and sixty-two (62.8%) of the study participants had a history of contraceptive use prior to their current pregnancy (Table 2).

**Table 2:** Lifestyle, clinical, and obstetric-related characteristics of pregnant women attending ANC units at governmental health institutions in Gondar city administration, Northwest Ethiopia, 2020 (n = 417)

Variables	Frequency	Percentage (%)
**Parity**		
Nulli & primi para	257	61.6
Multi & grand multipara	160	38.4
**Trimesters of gestation**		
1st & 2nd TM	147	35.3
3rd TM	270	64.7
**History of DM**		
Yes	15	3.6
No	402	96.4
**Gestational diabetes mellitus**		
Yes	19	4.6
No	398	95.4
**History of hypertension**		
Yes	22	5.3
No	395	94.7
**Pregnancy-induced hypertension**		
Yes	29	6.9
No	388	93.1
**History of vision problem**		
Yes	37	8.9
No	380	91.1
**Regular use of smartphones**		
Yes	245	58.7
No	172	41.3
**Regular use of computer or television**		
Yes	201	48.2
No	216	51.8
**Medication history**		
Yes	45	10.8
No	372	89.2
**Contraceptive use**		
Yes	262	62.8
No	155	37.2
**Sleep duration**		
Short	49	11.7
Optimal	263	63.1
Long	105	25.2
**Ever drink alcohol**		
Yes	284	68.1
No	133	31. 9
**Currently drink alcohol**		
Yes	183	64.4
No	101	35. 6
**Ever drink coffee**		
Yes	354	84. 9
No	63	15.1
**Currently drink coffee**		
Yes	289	81.6
No	65	18.4
**Perceived stress level**		
Low	75	18.0
Moderate	309	74.1
High	33	7.9

DM = Diabetes mellitus, TM = Trimester

### Prevalence of visual impairment and its associated factors

In the current study, the prevalence of VI among pregnant women was 22.5% (95% CI: 18.5–26.6). Thirty (7.2%) and thirty-two (7.7%) of the study participants had moderate to severe visual impairments in their right and left eyes, respectively. Of the total pregnant women who were visually impaired, 30 (7.2%) of them had bilateral VI and 64 (15.3%) had monocular VI.

Among all the variables entered into a binary logistic regression, age of the participants, residence, educational status, parity, gestational age, history of DM, GDM, history of HTN, PIH, regular use of computers or watching television and smartphones, medication history, history of contraceptive use, and sleep duration were associated with VI at p-value < 0.25. However, in the final model, only age, parity, gestational age and history of contraceptive use were significantly associated with VI at *p*-value ≤0.05.

Pregnant women aged 31–49 years had 2.1 times (AOR = 2.1, 95% CI: 1.1–4.0) higher odds of developing VI than those aged 15–30 years. Pregnant women with the third trimester of gestational age had 2.4 times (AOR = 2.4, 95% CI: 1.3–4.5) increased odds of VI than those with first and second trimesters of gestational age. Being multi & grand multiparous among pregnant women was 2.3 times (AOR = 2.3; 95% CI: 1.2–4.6) more likely to develop VI than those who were nulli and primiparous. The odds of having VI among pregnant women who had a history of contraceptive use before their current pregnancy was 2.7 times (AOR = 2.7; 95% CI: 1.2–6.3) higher than the non-users (Table 3).

**Table 3:** Bivariable and multivariable logistic regression analysis of factors associated with visual impairment among pregnant women attending ANC units at governmental health institutions in Gondar city administration, Northwest Ethiopia, 2020 (n = 417)

Variables	Visual impairment Yes N (%) No N (%)		OR (95% CI)	
			COR	AOR
**Age (years)**				
15–30	48 (15.5)	262 (84.5)	1.0	1.0
31–49	46 (43.0)	61 (57.0)	4.1 (2.5–6.7)	2.1 (1.1–4.0)*
**Residence**				
Urban	68 (19.7)	277 (80.3)	1.0	1.0
Rural	26 (36.1)	46 (63.9)	2.3 (1.3–4.0)	1.3 (0.6–3.0)
**Educational status**				
Can’t read & write	20 (37.0)	34 (63.0)	1.0	1.0
Primary	23 (24.2)	72 (75.8)	0.5 (0.3–1.1)	0.9 (0.4–2.1)
Secondary	10 (8.8)	103 (91.2)	0.2 (0.1–0.4)	0.5 (0.2–1.4)
College/University	41 (26.5)	114 (73.5)	0.6 (0.3–1.2)	1.1 (0.4–2.7)
**Trimesters of gestation**				
1st & 2nd TM	19 (12.9)	128 (87.1)	1.0	1.00
3rd TM	75 (27.8)	195 (72.2)	2.6 (1.5–4.5)	2.4 (1.3–4.5)*
**Parity**				
Nulli & primi para	27 (10.5)	230 (89.5)	1.0	1.0
Multi & grand multipara	67 (41.9)	93 (58.1)	6.1 (3.7–10.2)	2.3 (1.2–4.6)*
**History of DM**				
Yes	8 (53.3)	7 (46.7)	4.2 (1.5–11.9)	0.9 (0.2–4.5)
No	86 (21.9)	316 (78.1)	1.0	1.0
**Gestational DM**				
Yes	11 (57. 9)	8 (42.1)	5.2 (2.0–13.4)	2.1 (0.5–9.3)
No	83 (20.9)	315 (79.1)	1.0	1.0
**History of HTN**				
yes	9 (40.9)	13 (59.1)	2.5 (1.0–6.1)	1.2 (0.3–3.9)
No	85 (21.5)	310 (78.5)	1.0	1.0
**PIH**				
Yes	13 (44.8)	16 (55.2)	3.1 (1.4–6.7)	0.7 (0.3–2.2)
No	81 (20.9)	307 (79.1)	1.0	1.0
**Sleep duration**				
Short	16 (32.7)	33 (67.3)	4.6 (1.9–11.2)	1.9 (0.7–5.4)
Optimal	68 (25.9)	195 (74.1)	3.3 (1.6–6.7)	1.72 (0.8–3.8)
Long	10 (9.5)	95 (90.5)	1.0	1.0
**Regular use of smartphones**				
Yes	41 (16.7)	204 (83.3)	0. 5 (0.3–0.8)	1.3 (0.6–2.8)
No	53 (30.8)	119 (69.2)	1.0	1.0
**Regular use of a computer or TV**				
Yes	40 (19.9)	161 (80.1)	0.75 (0.5–1.2)	0.9 (0.5–1.8)
No	54 (25.0)	162 (75.0)	1.0	1.0
**History of medication**				
Yes	19 (42.2)	26 (57.8)	2.9 (1.5–5.5)	1.6 (0.7–3.7)
No	75 (20.2)	297 (79.8)	1.0	1.0
**History of contraceptive use**				
Yes	84 (32.1)	178 (67.9)	6.9 (3.4–13.7)	2.7 (1.2–6.3)*
No	10 (6.4)	145 (93.6)	1.0	1.0

AOR = Adjusted odds ratio, CI = confidence interval, COR = crude odds ratio, TM = Trimester, TV = Television, * = p-value ≤ 0.05

## Discussion

Major ocular changes are frequently observed in women of reproductive age groups, which occur more often during the time of their pregnancy. Most of these alterations during pregnancy are due to non-threatening physiological responses to the hormonal and metabolic adjustments to adopt the gestational product. However, there can be some critical pathological complications that may persist after the postpartum period. To the best of our knowledge, very little is known about the magnitude of VI among pregnant women in the world including Ethiopia. Thus, this research (the first of its kind in Ethiopia) tried to offer insight on the magnitude of VI and its associated factors among pregnant women attending ANC units at health institutions in Ethiopia, the case of Gondar city administration governmental health institutions.

In this study, the overall prevalence of visual impairment was 22.5% (95% CI: 18.5–26.6). Age, gestational age, parity, and history of contraceptive use were significantly associated with visual impairment (*p* ≤ 0.05).

Despite similar study population (pregnant women), the prevalence of VI among pregnant women in our study is lower than the previous studies conducted in Iran (89.2%) [8], India (65%) [33], South India (77.5%) [34], and USA (25–50%) [12]. This discrepancy might be due to the differences in study settings and study design. For example, we employed a cross-sectional study while most other studies used observational prospective studies. Another possible justification for the variation could be cultural and socio-economic characteristics of the study population, in which the population of Ethiopia, including women, have very little exposure to the potential risk factors like access to use digital devices and environmental hazards released from industries compared to the population in developed countries.

The prevalence of VI among pregnant women in our study is higher than previous studies (conducted on non-pregnant women) in India (12%) [11], Sub-Saharan Africa (4.17%) [5], South Africa (10.8%) [35], and Ethiopia (4.1%) [6]. This variation might be accounted for the differences in the study population. This means that the study population in our study were only pregnant women whereas in the above compared studies, the prevalence was among all adult females (non-pregnant). Hence, the higher prevalence of VI in our study population might be due to pregnancy-associated metabolic and hormonal changes, as justified by other study [14]. During pregnancy, there is elevation of levels of estrogen and progesterone, which trigger fluid retention in the cornea. As a result, this condition leads to corneal oedema, thickness and curvature, amplified lens thickness and temporary loss of accommodation, which subsequently impairs the normal refractive power of the eye and ends up with VI [14].

Other possible causes of VI in pregnant women were associated with neuro-ophthalmic and other pre-existing conditions precipitated by gravidity. During pregnancy, pre-existing diseases like Graves’ disease and Optic neuritis are exacerbated [14]. Diabetic retinopathy and central serous chorioretinopathy with an increased risk of retinal detachment can be worsened while pregnancy in women with pre-existing DM [14, 35] Neuro-ophthalmological disorders such as venous sinus thrombosis**,** benign intracranial hypertension**,** pituitary adenoma**,** and meningioma are more likely to be aggravated by gestational related changes in a woman during her pregnancy [14]. Pre-eclampsia and eclampsia, which may occur after 20 weeks of gestation, could result in hypertensive retinopathy, exudative retinal detachment, and cortical blindness [3, 14].

Consistent with the studies conducted in South Africa [35], Taiwan [36], Ghana [37], and Ethiopia [24], in this study, those women aged 31–49 years had higher odds of VI. This could be due to the increased risk of age-related diseases of the eye following ageing. As age increases, the nature and functions of the lens and cornea gradually decrease and this strongly affects the normal focusing of the light on the retina [14].

The finding of our study revealed that VI was more likely to occur in the women during the third trimester of pregnancy. This result is consistent with other studies in Turkey [38], India [34], Iran [39], and Nigeria [7, 22]. The possible reason for this incident could be due to the hormonal and metabolic changes as a result of gestational stress, which may lead to corneal thickness and alterations in the refractive power of the lens. These ocular changes in turn bring about VI among pregnant women [14].

Multi and grand-multiparous pregnant women were more liable to develop VI than those who were nulli and primiparous. This finding is supported by a previous study in the USA [15], Croatia [23], and China [20]. This event is probably accounted for the repeated ocular alteration in the succeeding gravidity of mothers who had a greater number of parity.

The odds of developing VI among pregnant women who had a history of contraceptive use prior to their current pregnancy was found to be higher than their counterparts. This finding is in agreement with studies in Iran [40], Germany [14], Egypt [32], and Greece [41]. This can be due to the fact that using contraceptives (oral and injectable) for family planning methods will cause dry eye symptoms related to reduced lipid synthesis, corneal oedema, and a significant increase in the central corneal thickness associated with the hormonal effects (estrogen and progesterone) [42, 43]. Occlusion of the central retinal artery, intraocular haemorrhages, macular or disc oedema, and acute ischemic optic neuropathy are also reported in women using contraceptive pills which may impair the normal visual pathway [42].

A reasonably high response rate (98.6%**)** could be the strength of our study. This study has also epidemiological data on the prevalence of visual impairment among pregnant women, which is the first institution-based study, not yet reported in Ethiopia. However, our study was based on an institution based cross-sectional study design, which can’t show the real cause-effect relationship between independent variables and VI, inferring that, we are not sure about the presence of VI before pregnancy and whether it is worsened or improved by the pregnancy itself. Another limitation of our study was that we didn’t collect information regarding the treatment status of the study participants with VI (whether they were actually treated or planned to be treated). Lastly, we didn’t identify the possible causes of VI because of limited resources.

## Conclusion

The magnitude of VI among pregnant women was high. It was significantly associated with maternal age of 31–49 years, gestational age in the 3rd trimester, multi & grand multiparous women, and those who had a history of contraceptive use before the current pregnancy. Since the prevalence of VI is high in our study, it requires a routine screening and evaluation of pregnant women at antenatal care visits. Further analytical investigations of visual changes, particularly as a result of pregnancy, could also be warranted.

## Data Availability

All pertinent data are comprised in the manuscript. The dataset is available and can be obtained from the corresponding author upon reasonable request.

## Abbreviations

ANC: antenatal care
AOR: Adjusted odds ratio
CI: confidence interval
DM: diabetes mellitus
GDM: gestational diabetes mellitus
PIH: pregnancy-induced hypertension
PVA: presenting visual acuity
VI: Visual Impairment
